# A 100-million-year old predator: a fossil neuropteran larva with unusually elongated mouthparts

**DOI:** 10.1186/s40851-019-0144-0

**Published:** 2019-08-30

**Authors:** Joachim T. Haug, Patrick Müller, Carolin Haug

**Affiliations:** 1 Ludwig Maximilians University Munich, Biocenter, Großhaderner Str. 2, 82152 Planegg, Martinsried Germany; 2 GeoBio-Center at LMU, Richard-Wagner-Str. 10, 80333 Munich, Germany; 3 Käshofen, Germany

**Keywords:** Neuroptera, Lacewings, Burmese amber, Cretaceous, Convergent evolution

## Abstract

**Background:**

Biological diversity is a hot topic in current research, especially its observed decrease in modern times. Investigations of past ecosystems offer additional insights to help better understand the processes underlying biodiversity. The Cretaceous period is of special interest in this context, especially with respect to arthropods. During that period, representatives of many modern lineages appeared for the first time, while representatives of more ancient groups also co-occurred. At the same time, side branches of radiating groups with ‘experimental morphologies’ emerged that seemed to go extinct shortly afterwards. However, larval forms, with their morphological diversity, are largely neglected in such studies, but may provide important insights into morphological and ecological diversity and its changes in the past.

**Results:**

We present here a new fossil insectan larva, a larval lacewing, in Cretaceous amber, exhibiting a rather unusual, ‘experimental’ morphology. The specimen possesses extremely large (in relation to body size) mandibulo-maxillary piercing stylets. Additionally, the labial palps are very long and are subdivided into numerous elements, overall appearing antenniform. In other aspects, the larva resembles many other neuropteran-type larvae.

**Conclusions:**

We provide a comparison that includes quantitative aspects of different types of neuropteran larvae to emphasise the exceptionality of the new larva, and discuss its possible relationships to known lineages of Neuroptera; possible interpretations are closer relationships to Dilaridae or Osmylidae. In any case, several of the observed characters must have evolved convergently. With this new find, we expand the known morphological diversity of neuropterans in the Cretaceous fauna.

## Background

Reconstructing changes in past biodiversity has become an important field of research. In the content of severe modern-day biodiversity losses, we hope to understand, and possibly influence, modern losses by investigating comparable processes in the past [1, 2].

The Cretaceous period (145–66 mya) has become a kind of “hot spot” time period concerning biodiversity, not only because it ended with a dramatic mass extinction that terminated the era of the large dinosaurs. Three factors made the Cretaceous an extremely diverse period especially among arthropods, i.e. insects, crustaceans, chelicerates, and their relatives, a dominating group of animals in all ecosystems now and in the past:

A.) The appearance and diversification of many lineages with nowadays abundant, well-known representatives, such as ants, bees, termites, or crabs, but also of less well-known groups, such as modern-type slipper lobsters [3–9].
B.) The survival of older groups, possessing morphologies still known from Palaeozoic times (ending ca. 252 mya), but being extinct in later faunas. This includes, for example, very early offshoots of the evolutionary lineage of mayflies [10] and cockroaches [11].
C.) Early side branches of radiating groups with “experimental morphologies” that subsequently became extinct (e.g., Alienoptera: early representatives of the lineage towards mantises [12]; Tarachoptera: early relatives of caddisflies and butterflies [13]; Haidomyrmecini: ants distantly reminiscent of trap-jaw ants [14]).

These three factors appear to have led to a very diverse fauna in the Cretaceous; very different morphological and ecological strategies of different representatives within a single group co-occurred within a single fauna [11]. And yet we likely miss larger parts of the period’s true diversity by restricting our view to taxonomic questions, i.e. presence of specific lineages, old ones, new ones, side branches etc.

What we miss in such an approach is, for example, the diversity of larval forms (for the challenges of the term larva, see [15]). Larvae are trickier to deal with from the taxonomic aspect, i.e., to identify them to a narrower systematic group is often difficult. Yet, especially for Holometabola (such as bees, beetles and butterflies) the larval phase of their lives appears to have the larger ecological impact, as this phase lasts longer and many individuals do not reach adulthood.

In the group Neuroptera, lacewings, spectacular larval forms are known in modern fauna, such as antlions. Nonetheless, Cretaceous fossils described in recent years have demonstrated that 100 million years ago even more and stranger appearing forms have lived [16–18].

Here we report another unusual neuropteran larva from 100 million years old Cretaceous Burmese amber (for geological information on Burmese amber, see [19, 20]). The larva is challenging to treat in a systematic and taxonomic context, but exhibits a previously unknown morphology.

## Material and methods

### Material

**Fig. 1:**
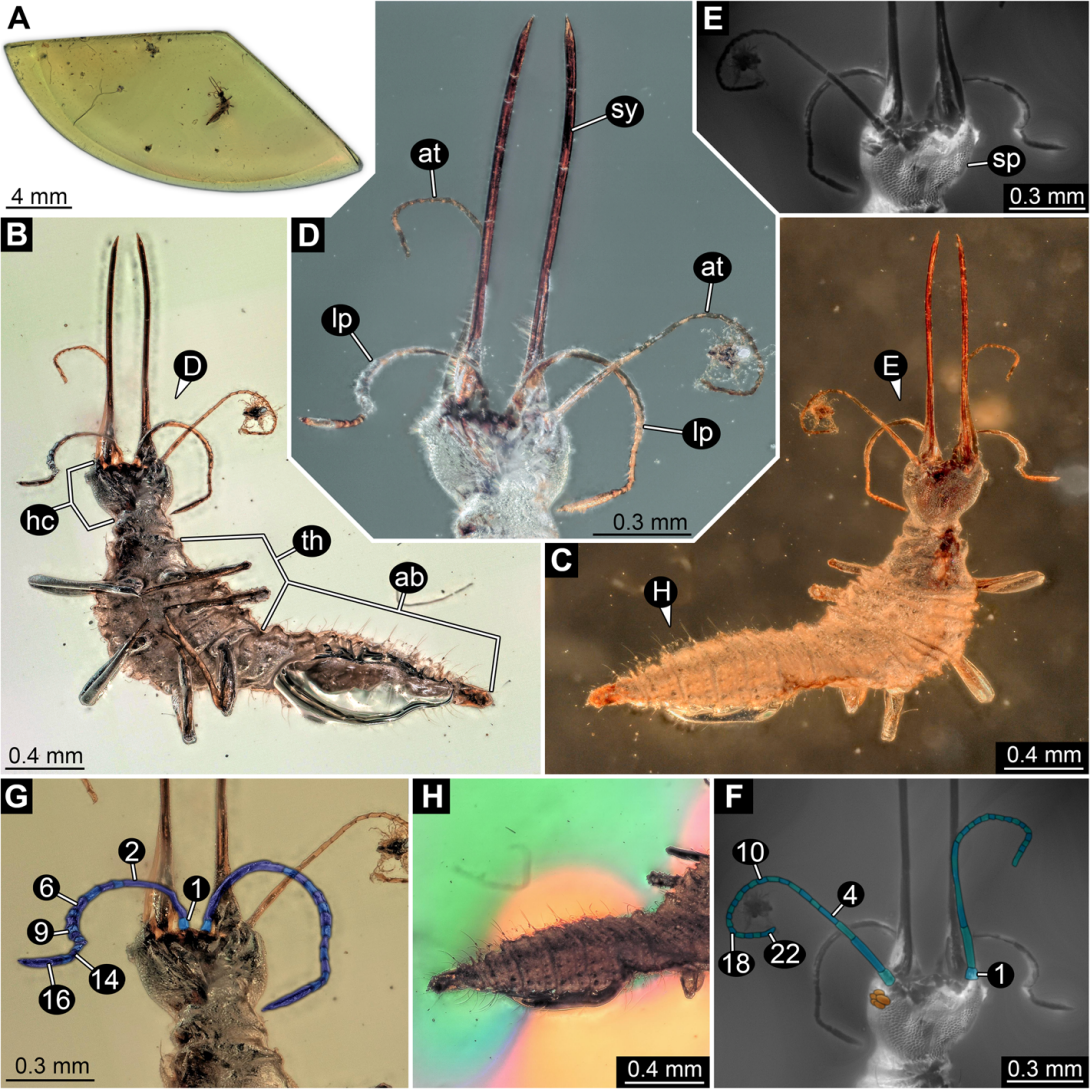
Overview and details of the new neuropteran larva (SMNS BU-355) with large stylets. **a**–**d**. Composite white-light micrograph. **a**. Overview image of the amber piece. **b**–**d**. Ring illumination, reflective. **b**. Overview of specimen in ventral view (white background). **c**. Overview of specimen in dorsal view (black background) **d**. Close up of head in ventral view (black background). **e**, **f**. Composite auto-fluorescence micrographs; dorsal view on head. **e**. Native. **f**. Structures highlighted by colour-markings; stemmata in orange, subdivision of antennae in blue and cyan. **g**, **h**. Composite white-light micrographs. **g**. Ventral region of head (white background); labial palp subdivision highlighted by colour-markings. **h**. Posterior trunk (abdomen) under coaxial cross-polarised light, providing more contrast for recognising folds and setae. Colour in background is side effect of polarisation. Abbreviations: 1–22 = number of elements; ab = abdomen; at = antenna (antennula in neutral arthropod terminology); hc = head capsule; lp = labial palp (maxillary palp in neutral arthropod terminology); sp. = scale-like pattern; sy = mandibulo-maxillary stylet; th = thorax

A single larval neuropteran specimen preserved in Burmese amber was investigated (Fig. 1a). The specimen originates from the Hukawng Valley, Kachin State, Myanmar and was part of the private collection of one of the authors (PM) under the repository number BUB 2943. It is now part of the collections of the Staatliches Museum für Naturkunde Stuttgart (“Löwentormuseum”) under repository number SMNS BU-355.

The raw amber piece was first cut with a Dremel 3000. Afterwards it was polished with wet sandpaper, first grade 200, and then subsequently grade 600, 1000 and 5000. The final polishing was performed with Sidol metal polish.

### Documentation

The specimen was documented with composite imaging under different white light conditions, as well as under autofluorescence. The white-light microscopic images were recorded with a Keyence VHX-6000 equipped with a 20–2000 × objective, either under ring illumination or under coaxial cross-polarised illumination. Black and white background colour was used. To achieve an optimal result, every image was recorded with different exposure times (HDR) [18, 21]. For autofluorescence images, a Keyence BZ-9000 was used [22, 23].

Each image detail was documented as a stack, with the single images of the stack (frames) being recorded in different focal levels in z-axis to overcome limitations in depth of field. The frames of each stack were fused to achieve an entirely sharp image. Several adjacent stacks were recorded in x-y axis to overcome limitations in field of view. All image details were stitched to a final panorama image [24, 25].

Additionally, based on the stacks the three-dimensional relief information was extracted (virtual surface). This information is presented as red-cyan stereo anaglyphs [26].

Drawings of the specimen and of comparative material were prepared in Adobe Illustrator CS2.

### Measurements

Different morphological dimensions of the new specimen as well as of different fossil and extant neuropteran larvae depicted in scientific publications were measured. These measurements include: body length (excluding mandibles), head width, trunk width, mandible length (direct line from proximal joint to distal tip), and labial palp length (along the outline as it is flexible). From the resulting values ratios were calculated (Table 1), as often no scales were available. The ratios were plotted into scatter plots to illustrate the rough body shape, the relative length of the mandibles, and the relative length of the labial palps. The last value (labial palp length) was only obtained for groups without lacking (Sisyridae) or very short (i.e., Myrmeleontiformia) labial palps, approximately corresponding to half of the measured specimens.

**Table 1:** Ratios of measured dimensions of different neuropteran larvae for scatter plots in Fig. 3

Group	Species	Larval instar	Source	Fig. in source	Occurrence	body length / trunk width	head width / trunk width	mandible length / body length	labial palp / body length
New fossil larva	–		This paper		Burmese amber	3.59	0.56	0.46	0.39
Ascalaphidae	*Ascaloptynx furciger*	3	[27]	5	extant	1.97	0.44	0.25	0.00
Ascalaphidae	*Ascaloptynx furciger*	1	[27]	7	extant	2.65	1.10	0.54	0.00
Ascalaphidae	*Haploglenius* sp.		[28]	8A	extant	2.43	0.47	0.21	0.00
Ascalaphidae	*Libelloides* sp.	3	[29]	A-84	extant	2.26	0.65	0.26	0.00
Ascalaphidae	*Puer maculatus*		[28]	8B	extant	1.76	0.41	0.23	0.00
Ascalaphidae	*Ululodes mexicana*	3	[27]	1	extant	2.11	0.43	0.25	0.00
Ascalaphidae	*Ululodes mexicana*	1	[27]	4	extant	2.68	1.06	0.59	0.00
Ascalaphidae	*Ululodes* sp.		[30]	9.24	Dominican amber	1.77	0.83	0.62	0.00
Ascalaphidae	–		[30]	9.22	extant	2.08	0.63	0.30	0.00
Berothidae	*Lomamyia* sp.	1	[31]	2	extant	9.22	0.89	0.13	0.08
Berothidae	*Podallea vasseana*	1	[32]	7	extant	6.92	0.92	0.17	0.14
Berothidae	*Spermophorella* sp.	1	[31]	4	extant	7.21	0.71	0.12	0.07
Berothidae	–		[33]	6 L	extant	7.88	0.29	0.04	0.04
Chrysopidae	*Berchmansus adumbratus*	3	[34]	1H	extant	4.50	0.50	0.09	0.07
Chrysopidae	*Chrysopa pallens*		[35]	S.27 u.	extant	3.11	0.31	0.14	0.08
Chrysopidae	–		[33]	6C	extant	4.35	0.38	0.09	0.07
Coniopterygidae	*Coniopteryx* sp.	3	[29]	C-173	extant	3.10	0.48	0.07	0.10
Coniopterygidae	*Conventzia pineticola*	3	[36]	3	extant	4.29	0.54	0.07	0.10
Coniopterygidae	*Conventzia psociformis*	3	[36]	4	extant	3.24	0.35	0.06	0.08
Dilaridae	*Nallachius* sp.	3	[31]	6	extant	11.42	0.50	0.05	0.04
Dilaridae	–		[33]	6I	extant	10.75	0.33	0.04	0.03
Hemerobiidae	*Hemerobius pini*		[29]	H-10	extant	4.40	0.50	0.09	0.07
Hemerobiidae	*Mecromus vagus*	3	[37]	1	extant	5.07	0.41	0.03	0.04
Ithonidae	*Oliarces clara*	1	[38]	23	extant	–	–	0.12	0.14
Mantispidae	*Ditaxis biseriata*	1	[39]	3	extant	6.25	0.69	0.12	0.09
Mantispidae	*Mantispa syriaca*	1	[29]	M-10	extant	6.00	0.50	0.06	0.10
Myrmeleontidae	*Euroleon nostras*		[28]	8D	extant	1.94	0.36	0.23	0.00
Myrmeleontidae	*Myrmeleon inconspicuus*	3	[29]	M-60	extant	2.21	0.32	0.19	0.00
Myrmeleontidae	*Tricholeon relictus*		[28]	8C	extant	2.54	0.46	0.16	0.00
Myrmeleontidae	–		[33]	5G	extant	2.52	0.52	0.22	0.00
Nemopteridae	*Amerocroce boliviana*		[40]	15	extant	3.26	0.49	0.12	0.00
Nemopteridae	*Laurhervasia setacea*	3	[30]	9.17	extant	3.55	0.65	0.15	0.00
Nemopteridae	*Moranida peruviensis*		[40]	14	extant	3.21	0.35	0.15	0.00
Nemopteridae	*Veuriese bruchi*		[40]	25	extant	2.62	0.40	0.17	0.00
Nemopteridae	–		[33]	5B	extant	2.09	0.50	0.12	0.00
Nevrorthidae	*Austroneurortus* sp.		[41]	1	extant	–	–	0.13	0.08
Nevrorthidae	*Nevrorthus fallax*		[30]	9.13	extant	11.65	0.59	0.08	0.06
Nevrorthidae	?*Rophalis relicta*	1	[42]	07.21 b	Baltic amber	3.96	0.92	0.24	0.26
Nevrorthidae	–		[33]	4	extant	9.73	0.55	0.08	0.07
Nevrorthidae	–		[42]	07.18 a	Baltic amber	7.90	0.44	0.08	0.06
Nymphidae	–		[33]	5F	extant	2.84	0.59	0.23	0.00
Nymphidae	–		[33]	5E	extant	1.24	0.38	0.36	0.00
Osmylidae	*Osmylus fulvicephalus*		[43]	43	extant	3.79	0.61	0.32	0.17
Osmylidae	*Osmylus fulvicephalus*		[43]	45	extant	5.95	0.37	0.16	0.00
Osmylidae	*Osmylus fulvicephalus*		[35]	S.26 u.l.	extant	4.86	0.48	0.16	0.09
Osmylidae	*Osmylus fulvicephalus*	3	[29]	O-30	extant	5.06	0.63	0.23	0.12
Osmylidae	*Osmylus fulvicephalus*	1	[29]	O-30	extant	3.92	0.83	0.49	0.13
Osmylidae	*Osmylus fulvicephalus*		[30]	9.26	extant	5.06	0.61	0.16	0.08
Osmylidae	–		[33]	6D	extant	8.29	0.71	0.21	0.11
Osmylidae	–	1	[42]	07.04 a	Baltic amber	3.62	1.00	0.51	0.23
Osmylidae	*Osmylus fulvicephalus*	2	[44]	4	extant	4.68	1.00	0.40	0.26
Polystoechotidae	–	1	[38]	30	extant	–	–	0.09	0.08
Psychopsidae	–		[33]	5A	extant	3.93	1.07	0.29	0.00
Sisyridae	*Sisyra dalii*	2	[45]	64	extant	3.52	0.39	0.19	0.00
Sisyridae	*Sisyra dalii*	3	[45]	67	extant	2.81	0.30	0.28	0.00
Sisyridae	*Sisyra fuscata*		[35]	S.26 u.r.	extant	2.63	0.37	0.38	0.00
Sisyridae	*Sisyra iridipennis*	2	[45]	39	extant	3.88	0.42	0.29	0.00
Sisyridae	*Sisyra iridipennis*	3	[45]	43	extant	3.88	0.33	0.26	0.00
Sisyridae	*Sisyra jutlandica*	2	[45]	14	extant	3.80	0.50	0.25	0.00
Sisyridae	*Sisyra jutlandica*	3	[45]	21	extant	3.54	0.27	0.25	0.00
Sisyridae	*Sisyra* sp.		[29]	S-82	extant	3.69	0.23	0.31	0.00
Sisyridae	–		[33]	6G	extant	3.26	0.29	0.34	0.00

## Results

### Morphological description

#### General habitus

**Fig. 2:**
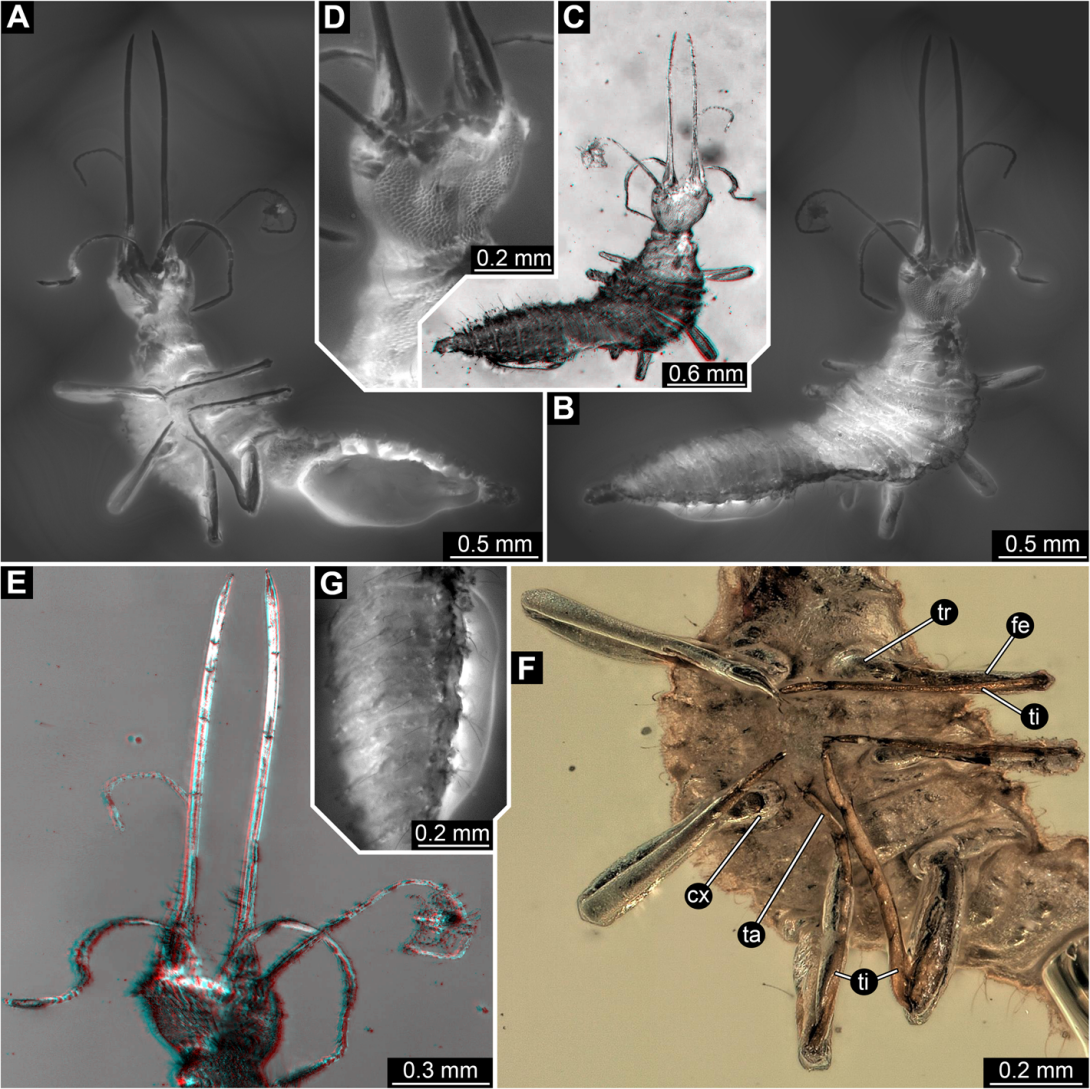
Overview and details of the new neuropteran larva (SMNS BU-355) with large stylets (continued). **a**, **b**, **d**, **g**, composite auto-fluorescence micrographs; **c**, **e**, stereo red-cyan-anaglyphs based on virtual surface reconstruction, colour-inverted; use red-cyan-glasses to view. **a**, overview of specimen in ventral view. **b**, overview of specimen in dorsal view. **c**, overview of specimen in dorsal view. **d**, close-up on surface of head and cervix, dorsal view. **e**, close-up on head, ventral view. **f**, composite white-light micrograph with ring illumination, reflective, white background; close-up on thorax with walking appendages; note long tibiae. **g**, close-up on abdomen; note setae on surface. Abbreviations: cx = coxa; fe = femur; ta = tarsus; ti = tibia; tr = trochanter

Small holometabolan larva, about 2.53 mm long (Figs. 1b, c, 2a–c). Body (presumably) organised into 20 segments. First body segment (ocular segment) and following five (post-ocular segments 1–5) forming distinct head with sclerotised head capsule. Trunk subdivided into two functional units. Anterior three trunk segments (post-ocular segments 6–8) sub-similar (thoracic segments) with prominent ventral appendages (thoracic appendages). Posterior eleven trunk segments without such appendages (Figs. 1h, 2g). Body without appendages about three times as long as maximum width. Maximum width at about 30% along the anterior-posterior axis.

#### Head region

Head capsule of sub-trapezoidal shape in dorsal (or ventral) view, dorso-ventrally flattened (Figs. 1d–g, 2d, e). Anterior edge of head capsule almost straight, about 0.46 mm wide, central third slightly drawn out anteriorly into a flat triangle. Posterior edge almost straight; only about 60% as wide as anterior edge, about 0.28 mm. Length of head capsule about as long as width of posterior edge. Lateral edges slightly bulging, not straight. Dorsal surface of head capsule covered with scale-like ornament, at least 35 loosely organised rows of such scale-like ornaments from one lateral edge to the other (Fig. 2d). Slight impression of a Y-shaped moult suture (mainly visible under fluorescence; Figs. 1e, 2d).

Ocular segment recognisable by larval eyes (stemmata). Stemmata located on a slight protrusion arising far anteriorly laterally from the head capsule (Fig. 1f). At least five individual stemmata per side. Stemmata larger than scale-like ornaments; diameter about as wide as three scale-like ornaments.

Post-ocular segment 1 recognisable by a pair of appendages, the antennae (antennulae in neutral arthropod terminology). Antennae arising from small hump far laterally from the anterior-dorsal region of the head capsule (Fig. 1f). Diameter at the base slightly larger than diameter of stemma. Antenna long, elongate, about three times as long as the length of the head capsule, subdivided into 22 articles. Proximal article about 1.5 times as long as wide (diameter). Article 2 slightly more slender and significantly longer, about three times as long as preceding element. Article 3 shorter, about 50% of preceding article, slightly more slender. Article 4 resembling article 3, only very slightly more slender. Article 5 to article 21 of sub-similar morphology. Article 5 about 50% of length of article 4, following articles slightly varying in size, in general, decreasing in length and also slightly in diameter. Distal article (22) about two times as long as preceding article, distally rounded.

Post-ocular segment 2 (intercalary segment) not externally recognisable.

Post-ocular segment 3 recognisable by a pair of appendages, the mandibles. Mandibles very long, elongate; about 50% of the main body proper length (Fig. 1b, d). Very gentle S-curvature in dorsal view slightly curving outside (laterally) proximally and inside (medially) distally. In most regions, about as wide as two stemmata, wider at the base. Strongly tapering at the tip, providing a sharp, syringe-like impression. A thin line along the mandible indicates a not directly observable groove at the functional ventral side (posterior side; Figs. 1d, 2e).

Post-ocular segment 4 recognisable by a pair of appendages, the maxillae (maxillulae in neutral arthropod terminology). Closely associated with the mandibles, forming mandible–maxilla complex, a functional stylet, about 1.20 mm long (Fig. 1d). Maxilla wider at base and this wider part reaching further distally than that of mandible; exact length not observable, most likely similar in length to mandible.

Post-ocular segment 5 recognisable by a pair of appendages, the labium, representing a functional coupled pair of originally individual appendages (maxillae or “second” maxillae in neutral arthropod terminology). Proximal region difficult to discern, based on the insertion of the distal parts, elongate, reaching to the anterior edge of the head. The distal parts, palps (endopods, “telopods”) are prominently developed. They are very elongate, and each is subdivided into at least 16 elements (Fig. 1g). Proximal element about as long as wide. Next distal region (counted as element 2) elongate curved, possibly either originally subdivided into 8–10 elements (based on length comparison) or to be subdivided during ontogeny. Element 3 longer than proximal element, hence slightly longer than wide. Following elements (4–15) all sub-similar to element 3. Distal element (16) about as long as four further proximal elements, tapering distally.

#### Trunk region

Transition of head to trunk with a distinct collar-like sclerite, cervix (Fig. 1b, g). Anterior edge slightly wider than posterior edge of head capsule, first widening and then tapering posteriorly, posterior edge about as wide as anterior edge, widest region in the middle. Length about 30% of width of anterior edge. The middle with a more or less distinct abaxial crest. Surface with an indication of scale-like ornament, yet much less distinct than on the head capsule.

All trunk segments with distinct dorsal and ventral surfaces, set off from anterior and posterior structures by distinct folds. Not distinctly sclerotised into easily detectable tergite and sternite. First three trunk segments with sub-similar morphology, forming a functional unit, thorax.

Post-ocular segment 6 (trunk segment 1), prothorax, wider than cervix at anterior edge, narrowing towards the mid region, but widening further posteriorly. Anterior edge about as wide as maximum width of head capsule, posterior edge wider than head capsule. Length about 50% of width of anterior edge. Surface with scale-like ornament similar to pattern of head capsule, more apparent towards the lateral. A single seta on each side anterior-laterally (Fig. 2b, d). Ventrally, close to the posterior edge a pair of prominent walking appendages, foreleg (thoracopod, Fig. 2f).

Foreleg composed of five individual elements. Most proximal element (coxa, most likely basipod in neutral arthropod terminology) ring-like in ventral view, tapering distally. More distal elements (endopod, “telopod”) significantly more slender than coxa, overall tube-shaped. Proximal endopod element (trochanter) short, curved outward, about as long as diameter of coxa, about two times as long as wide. Endopod element 2 (femur) more than three times as long as trochanter; diameter similar to that of trochanter. Endopod element 3 (tibia) very elongate and even more slender. Slightly longer than trochanter and femur combined, only about half the diameter of the trochanter; in anterior view very gently curved outwards. Distal endopod element (element 4, tarsus) not further subdivided. Shorter than trochanter, diameter similar to that of tibia. Distally carrying a pair of hook-shaped claws slightly longer than the diameter of the tarsus.

Post-ocular segment 7, mesothorax, wider than prothorax at anterior edge, widening posteriorly. Lateral edges with three distinct humps each. Lateral regions of dorsal side covered with scale-like ornament. Close to anterior edge is a row of about eight (estimated) smaller humps each carrying a seta (Fig. 2b). Closer to posterior edge is a row of about six such humps. Indistinct folds subdivide the dorsal region of the segment into roughly three regions. Ventrally, close to the posterior edge is a pair of prominent walking appendages, midleg, sub-similar to that of the prothorax (Fig. 2f).

Post-ocular segment 8 more difficult to observe, as the trunk is slightly distorted here. Appears slightly narrower than preceding segment, very slightly tapering posteriorly. Longer in anterior-posterior axis. Observable surface ornamentation resembling that of preceding segment, including scale-like ornament, lateral and dorsal humps with setae and folds. Ventrally close to the posterior edge is a pair of prominent walking appendages, hindleg, sub-similar to that of the prothorax, but slightly more elongated (Fig. 2f).

Post-ocular segments 9–19 (trunk segments 4–14) sub-similar, forming a functional unit, abdomen (Figs. 1h, 2g; Insecta-type abdomen not comparable to that of other euarthropods); only 10 segments externally visible; most likely segments 10 + 11 not differentiable from each other. Abdomen in anterior region slightly narrower than posterior edge of thorax, strongly tapering posteriorly, each segment is narrower than the next anterior segment; abdominal segment 1 is five times as wide as terminal segment (10 + 11). Abdomen further differentiated. Post-ocular segments 9–15 (abdominal segments 1–7) more similar to each other. Ventral region of abdomen largely obscured by bubble.

Post-ocular segment 9 (abdominal segment 1) shorter than metathorax, only about 30% of the length (anterior-posterior length). Segment indistinctly subdivided into three regions by abaxial folds (Fig. 2b). Each of the three regions with small humps of which most bear setae. Exact number difficult to discern, estimated 12 in anterior region, six in middle region, four in posterior region. Postero-laterally the segment bears a hump on each side from which socketed setae arise (exact number difficult, most likely two longer ones and one shorter one without distinct socket).

Post-ocular segment 10 (abdominal segment 2) sub-similar to preceding segment. Due to being slightly narrower fewer humps (approx. 11, 6, 4). In addition to the postero-lateral hump a smaller less distinct hump antero-laterally (on each side) with two (?) setae without distinct sockets.

Post-ocular segment 11 (abdominal segment 3) sub-similar to preceding segment. Due to being slightly narrower fewer humps (approx. 10, 6, 4). Postero-lateral hump more distinct, almost cone-shaped.

Post-ocular segment 12 (abdominal segment 4) sub-similar to preceding segment, due to being slightly narrower fewer humps (approx. 9, 4, 4).

Post-ocular segment 13 (abdominal segment 5) sub-similar to preceding segment, due to being slightly narrower fewer humps (approx. 8, 4, 2).

Post-ocular segment 14 (abdominal segment 6) sub-similar to preceding segment, due to being slightly narrower fewer humps (approx. 7, 2, 2).

Post-ocular segment 15 (abdominal segment 7) sub-similar to preceding segment, due to being slightly narrower fewer humps (approx. 6, 2, 2).

Post-ocular segment 16 (abdominal segment 8) with less distinct surface, appearing rough, but no apparent humps or similar. A single abaxial fold separates an anterior and a posterior region of the segment. Comparably to more anterior segments with two humps on each side, both almost cone-shaped, with a single socketed seta each.

Post-ocular segment 17 (abdominal segment 9) narrower, simple, no humps or folds. Laterally with two longer socketed setae, and one unsocketed shorter seta.

Terminal trunk element (most likely representing conjoined post-ocular segments 18 + 19, i.e. abdominal segments 10 + 11) simple terminally rounded, with five setae on each side.

### Results of scatter plots

**Fig. 3:**
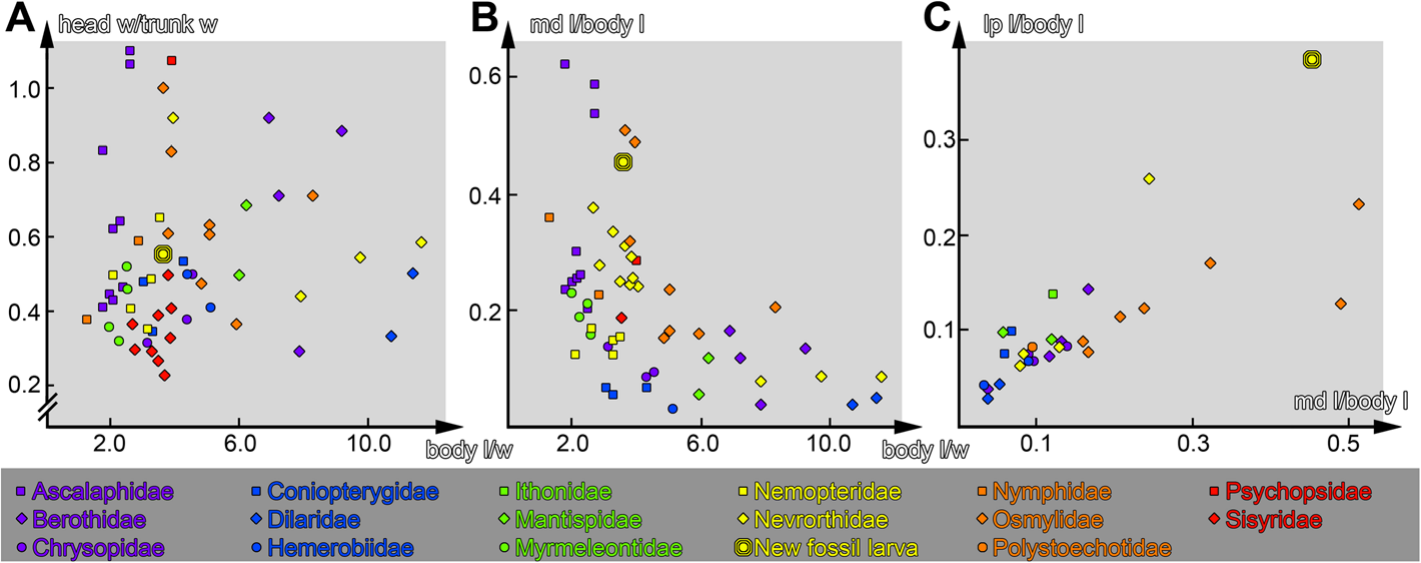
Scatter plots of ratios of measured dimensions of different neuropteran larvae (see Table 1). **a**, head width:trunk width ratio vs. body length:trunk width ratio; this plot describes the general body shape. **b**, mandible length:body length ratio vs. body length:trunk width ratio; this plot illustrates the relative length of the mandibles compared to the body dimensions. **c**, labial palp length: body length ratio vs. mandible length: body length ratio; this plot shows the relative lengths of mandibles and labial palps; note the eccentric position of the new larva

Concerning the general body shape, the new larva clusters among many other neuropteran larvae (Fig. 3a). However, concerning relative mandibular length the new larva clusters above the majority of the investigated neuropteran larvae, hence having relatively longer mandibles, but there are still other larvae clustering in the same area, all of them being first instar larvae (Fig. 3b, Table 1). When comparing the relative length of mandibles and labial palps, the new larva is clearly different from all other investigated neuropteran larvae (also of those in which labial palps are not lacking (Sisyridae) or very short (Myrmeleontiformia)). It does not only have relatively longer mandibles, but especially much longer labial palps (Fig. 3c).

## Discussion

### Phylogenetic interpretation: neuropteran relationships

The small larva can easily be identified as a neuropteran. The soft-appearing outer cuticle with three pairs of walking appendages, distinct head capsule and especially the stemmata (few simple eyes) immediately support an ingroup position within Holometabola. The prominent mandibles, apparently interconnected with parts of the maxilla, in a far anterior position (prognathous position of mouthparts), as well as a distinct collar-like connection between head and trunk (cervix) are autapomorphies of Neuroptera [41].

While an ingroup position of Neuroptera is easily supported by the observable characters, further-reaching conclusions are, as discussed in the following, more challenging. The ancestral state for the mouthparts of larvae in Neuroptera is a specialised mandible-maxilla complex, with mandibles and maxillae forming a pair of piercing-sucking structures or stylets, hence mandibulo-maxillary stylets [41, 46, 47]. To use these effectively, i.e., for piercing possible prey, a certain force is necessary, and furthermore an equal counterforce. Many neuropterans therefore have curved mandible-maxilla complexes that act against each other. In this way, force and counterforce are produced with the same mechanism; in fact a comparable mechanical solution is present in the like-wise piercing-injection type mouthparts of labidognathan spider or epimorphan centipedes. Yet, of course, here very different appendages have formed these counteracting mouthparts; the spider chelicerae correspond to the antennae of the neuropteran, the maxillipeds of the centipedes to the first walking appendages. Still this arrangement of counteraction orientation appears very effective.

The exact relationships of neuropterans are still a matter of debate [48]; review in [47]. The more traditional view interpreted Nevrorthidae (a species-poor group with relic distribution) as sister group to all other neuropterans. The remaining neuropterans were often further subdivided into Hemerobiiformia and Myrmeleontiformia. Alternatively, Myrmeleontiformia was interpreted as being nested inside Hemerobiiformia, the two branches Sisyridae and Osmylidae branching off before the split of Myrmeleontiformia and the rest of the neuropteran groups. The supposed sister group to Neuroptera, i.e., Megaloptera, and also the neuropteran in-groups Nevrorthidae and Sisyridae have fully aquatic larvae. Many larvae of Osmylidae can be considered as semi-aquatic. Based on this character distribution many authors have reconstructed the evolutionary history of Neuroptera with ancestrally aquatic larvae [42].

Yet, other phylogenetic reconstructions have drawn a distinctly different picture [49–51]. Here Coniopterygidae (in older reconstruction an ingroup of Hemerobiiformia) is the sister group to the remaining neuropterans. Nevrothidae, Sisyridae and Osmylidae form here a monophyletic group.

Besides these historical difficulties concerning the phylogeny of Neuroptera, it can additionally prove difficult to include larvae into phylogenetic reconstructions without knowing the adult form. Badano et al. [16] have demonstrated that it is well possible if important characters are available and provided a frame for Myrmeleontiformia. For Neuroptera as a whole, such a framework will still have to be developed. For the new specimen we discuss in the following key characters of the new larva that might provide a phylogenetic signal and compare it to known larval forms.

### Developmental state of the new specimen

**Fig. 4:**
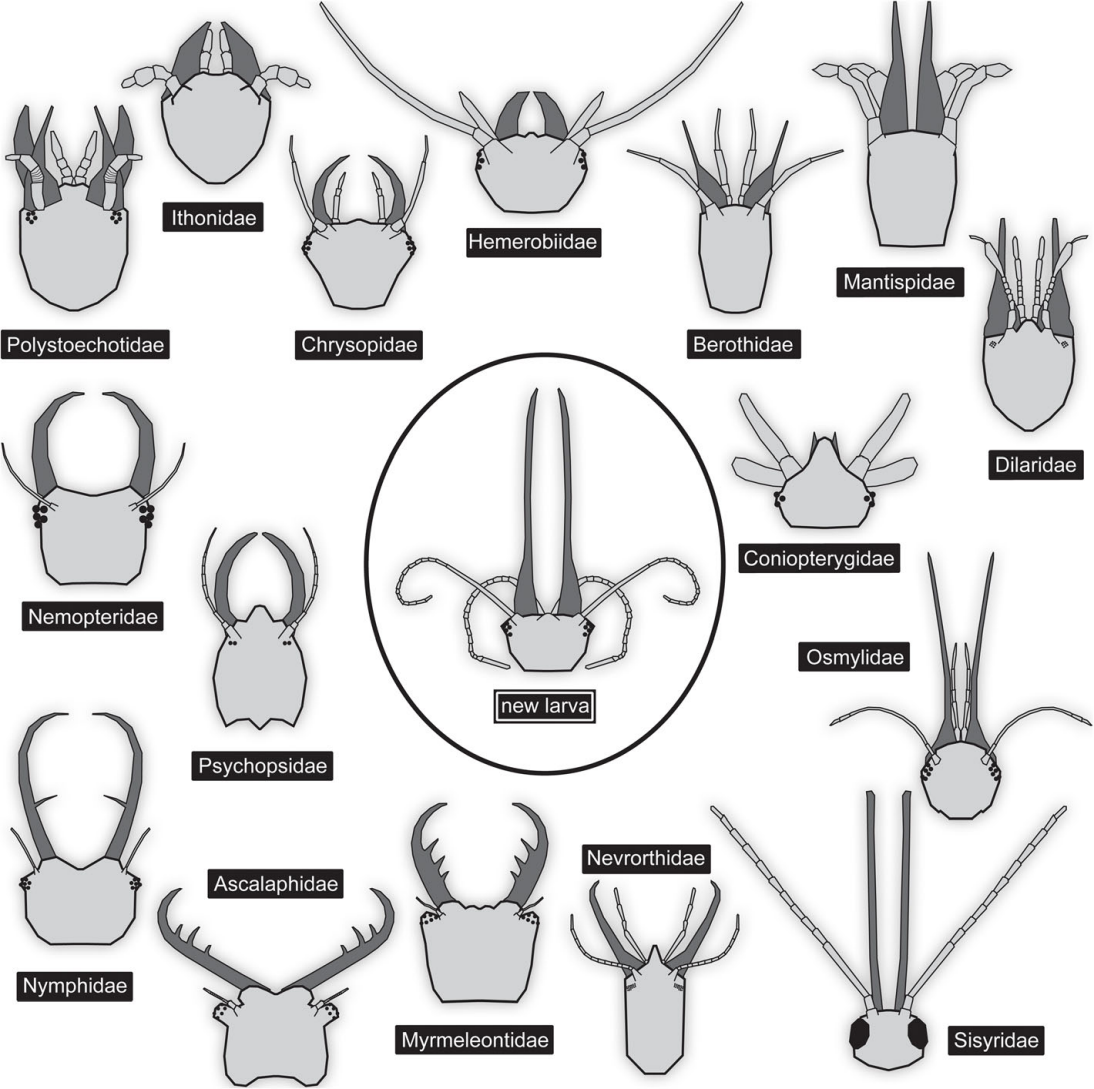
Comparison of the head of the newly described larva with larval heads of different groups of Neuroptera. Drawings simplified from various sources: Nevrorthidae: [41]; Myrmeleontidae: [28]; Ascalaphidae: [27]; Nymphidae: [52]; Psychopsidae: [33]; Nemopteridae: [40]; Polystoechotidae: [33]; Ithonidae: [38]; Chrysopidae: [34]; Hemerobiidae: [37]; Berothidae: [53]; Mantispidae: [39]; Dilaridae: [31]; Coniopterygidae: [36]; Osmylidae: [54]; Sisyridae: [45]

Before further interpreting the new larva we need to discuss the developmental state of the new fossil specimen in order to allow a proper comparison. Most neuropterans develop through three larval instars before transforming into the pupa (which will finally moult into the adult). The specimen seems unlikely to represent a first larval instar. The abdomen of the specimen is rather elongate and well developed. Many first instar larvae have a rather short abdomen, especially in relation to the thorax, as for example in larvae of Nevrorthidae and Osmylidae (Fig. 3b, c), and this tendency is also observed, for example, for larvae of Sisyridae [45] and Ascalaphidae ([27], his Fig. 4).

In first instar larvae, antennae and labial palps have significantly fewer subdivisions (e.g., in Sisyridae [45]; in Nevrorthidae [42]) and add further subdivisions in later larval instars [55]. In the new larva antennae and palps already possess numerous subdivisions. The relative length of the abdomen and the already far differentiated antennae and palps hence indicate that the new specimen is at least a larval instar 2. A further differentiation will depend on finding more specimens in different instars. Still it will be important for the further discussion that the new specimen is unlikely to represent a first instar larva.

### Phylogenetic interpretation: appearance of straight stylets

Despite uncertainty of neuropteran ingroup phylogeny (as outlined above), some distinct patterns can be identified (Fig. 4). For example, all representatives of Myrmeleontiformia (Myrmeleontidae, Ascalaphidae, Nymphidae, Psychopsidae, Nemopteridae) possess curved mandible-maxilla complexes as larvae, counteracting each other [41]. Curved mandible-maxilla complexes, i.e. stylets are also known in larvae of Nevrothidae (here the stylets are straight proximally and only curved distally, but still counteracting), and also in larvae of Hemerobiidae and Chrysopidae [33, 56, 57]. Less distinct, but clearly curved [38], are the stylets of larval forms of Ithonidae and Polystoechotidae.

Straight and forward directed stylets in which the two structures cannot counteract against each other, are known in larvae of various ingroups of Neuroptera. The larvae of Sisyridae (spongillaflies) are fully aquatic and possess long, very straight appearing, but in fact thin and flexible stylets. With these the larvae prey on (or parasitise?) sponges [47]. The larvae of Osmylidae (giant lacewings, lance lacewings) are often semi-aquatic, and possess even longer and more robust mandible-maxilla complexes that are slightly curving outwards [54].

While in these two groups the stylets are very long, in other cases in which straight stylets occur these are significantly shorter. The most extreme condition is found in larval forms of Coniopteryginae (in-group of dusty-wings). Here the stylets are so short that they are almost not visible in dorsal view [36]. Rather similar in overall appearance are the larger stylets in larvae of Dilaridae (pleasing lacewings; [58]), Berothidae (beaded lacewings; [53, 56]), Rhachiberothidae (thorny lacewings) and many Mantispidae (mantis lacewings; [39, 59]). Among the larvae of mantis lacewings, slightly curved stylets are also known [21, 60]. Depending on the exact phylogenetic reconstruction straight mandibles appear to have evolved independently at least three times.

### Comparison of the new fossil larva to modern larvae with straight stylets

The larva described here differs significantly from larval instars of Sisyridae. The head of sisyrid larvae is very small compared to the body (comparably so even in first instar larvae; [45], his fig. 9), the stylets are very slender, labial palps strongly reduced (factually absent) and the abdominal segments bear distinct gills (at least in second and third instar larvae; [45, 56, 57, 61]). In the new larva, the head is comparably larger, the stylets appear much more massive, labial palps are long and prominent, and there is no indication of abdominal gills (however, the ventro-abdominal region is largely obscured). Absence of gills may also be explainable by the new larva being a first instar, which could be compatible with its rather small size; yet, as pointed out above, the specimen is more likely at least an instar two larva.

**Fig. 5:**
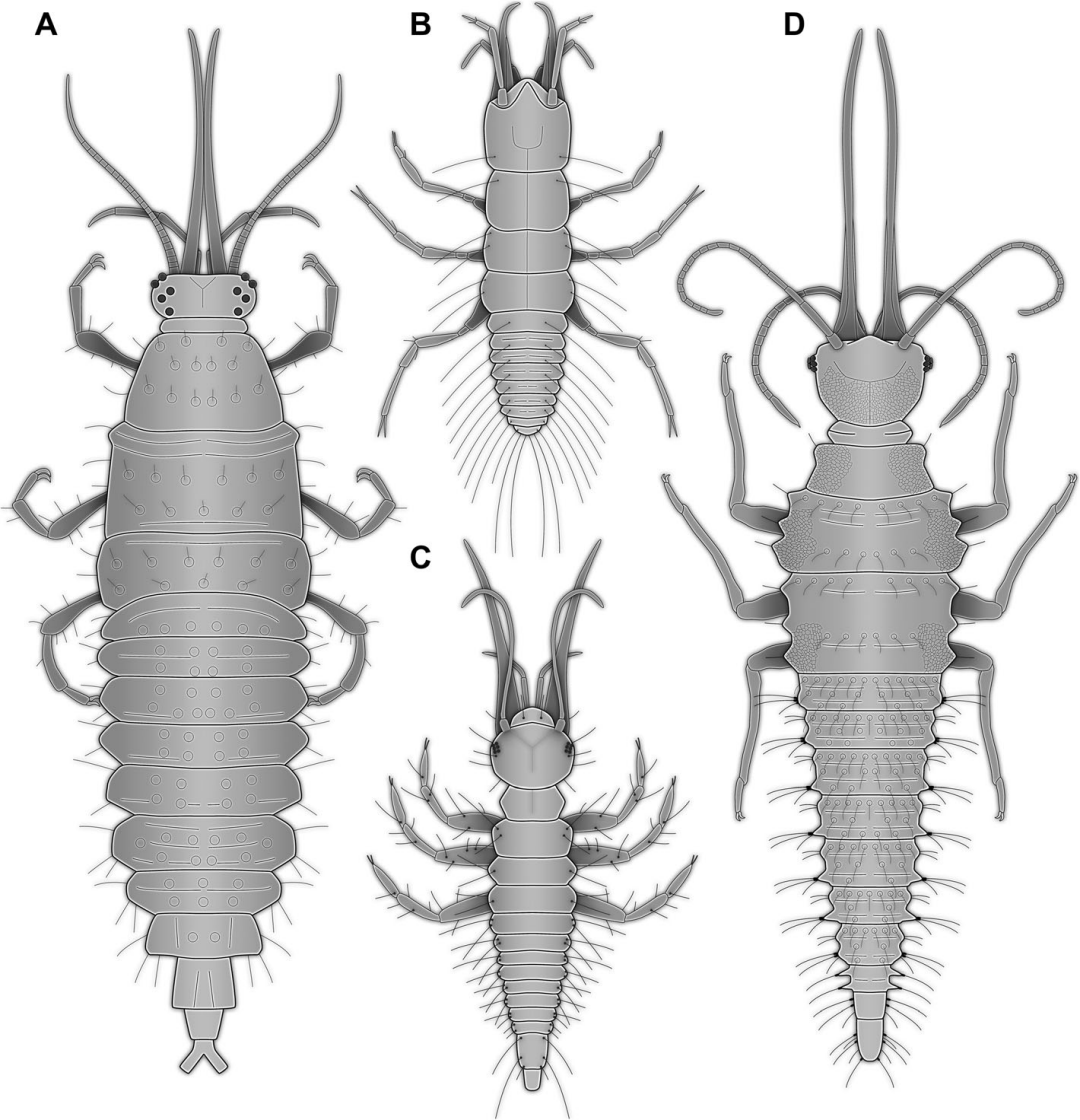
Different neuropteran larvae with mandibles relatively long compared to the body. **a**. Osmylidae (most likely larval instar 3; modified after Brauer 1851, in [43]). **b**. Nevrorthidae (combined from [42]). **c**. Osmylidae (larval instar 1; modified after [62]). **d**. Reconstruction of new larva (SMNS BU-355)

On a first sight, the new larva appears very similar to larvae of Osmylidae (Fig. 5). The head size, and especially the length and massiveness of the stylets, appear very similar. The relative length of the stylets decreases during larval ontogeny, as the abdomen becomes progressively longer (Fig. 3c; Table 1; [62]; osmylids, as neuropterans in general, have three larval instars; [44, 57, 63]). Only osmylids of instar one have in fact a stylet length similar to the condition in the new larva [62]. Already in instar two the abdomen has become longer and the ratio is below that of the new fossil. The new larva therefore has the relatively longest mandible-maxilla complexes for a larval instar two (and three).

There are also some more distinct differences between the new larva and osmylid larvae. The stylets of osmylid larvae are slightly curved outwards [54], while in the new fossil they are very slightly S-shaped with the tips being very slightly curved inwards. A further difference is the labial palp morphology; labial palps are significantly shorter and composed of much fewer elements in osmylid larvae [54]. Additionally, the legs of the new larva are significantly longer, especially the penultimate element (tibia) is much longer than the next proximal one (femur), i.e., the tibia is slightly longer than trochanter and femur combined, which is a rather unusual condition in neuropteran larvae and not present in osmylids. Also, osmylid larvae possess an empodium (a prominent trumpet-shaped structure arising between the tarsal claws), which appears not to be present in the larva described here.

In other groups with larvae with straight stylets these are, as already stated, significantly shorter. Nevertheless, the comparison is interesting as, at least in some, the labial palps are very prominent and have more subdivisions than in other groups (New [64] reports up to eight elements; see also [31], his fig. 11). Still, they have at most half as many elements as in the new larva, and also the enormous length as in the fossil is not reached.

Although the stylets of larvae of Nevrothidae are not straight, at least first instar larvae reach roughly comparable relative lengths of mandible-maxilla complexes and labial palps (Fig. 5). Also the tips of the mandibles of the new larva curve very slightly inwards, but not as distinctly as in nevrorthid larvae [41, 42, 55, 65]. The fossil larva differs significantly from larvae of Nevrorthidae in the shape of the head, prothorax and posterior body, which are all very slender and elongate in larval forms of Nevrothidae.

Although hemerobiid larvae differ significantly from the new larva in the stylet complex morphology, they share an important morphological aspect: the long tibiae of the thoracic appendages, very apparent in the new larva, which is a very uncommon character among neuropteran larvae, but is known in hemerobiid larvae [37].

### Phylogenetic interpretation summary

The new larva can hence not be easily identified as a representative of any of the already known groups within Neuroptera. It may well be a representative of the sister group to Osmylidae, before the outward curvature of the stylets evolved, the long and strongly subdivided labial palps and long tibiae representing specialisations of the new larva. Hence, it could be either interpreted as the earliest branch within Osmylidae or as sister group to Osmylidae.

Similarly simple in explanation, the new larva could be closely related to a group with shorter larval stylets, but strongly subdivided palps, as for example Dilaridae [31]. In this case the stylets, palps and tibiae would represent specialisations.

In both cases, we would need to assume a certain degree of convergence. Either the very long stylets or the high number of subdivision would need to be considered a case of convergence. In both cases the long tibiae would need to be understood as having evolved convergently to those in Hemerobiidae. Mixture of characters only explainable in the frame of convergence seems to be a common theme among fossil neuropteran larvae [16, 18].

### Taxonomy

Insecta Linnaeus, 1758

Holometabola Burmeister, 1835 (= Endopterygota Sharp, 1898)

Neuropteriformia Ax, 1999

Neuroptera Linnaeus, 1758

*incertae sedis*

Unnamed larva

Figures 1, 2 and 5 partim (drawing on the right).

### Ecological interpretation

Given the fact that the phylogenetic interpretation of the new larva is difficult, ecological interpretations of the new forms are also partly limited. A supposed closer relationship to Osmylidae could, for example, be indicative of a semi-aquatic lifestyle, further indicating a similar use of the stylets as in larvae of Osmylidae. In general, the functional morphology of the stylets of the new larva is difficult to understand. To pierce a prey item with such long and massive stylets requires a strong counteracting force, otherwise the new larva would simply push itself back when attempting to pierce its prey. The long tibia could be involved in providing a wider stance, making it possible to cling tightly to the surface anchoring the body. However, this suggestion must remain speculative. Similarly, the function of the long, strongly subdivided labial palp remains unclear, as we have no extant equivalent. In their overall appearance, the palps strongly resemble the likewise long antennae, providing the animal with something like two pairs of functional feelers.

As outlined in the introduction, the Cretaceous period is characterised by an enormous diversity caused by co-occurrence of already present modern forms, still present old forms, and new, but now extinct “experimental forms”. The new larva clearly falls into the third category. It possesses a unique morphology characterised by oversized piercing stylets and very long labial palps with an unusually high number of elements, twice as many as so far known, in combination with very long tibiae. It seems likely that the unusual length of three (mandibles, maxillae, labial palps) distinct structures (four, if we also count the comparably long antennae) has functional causes, i.e., these might be functionally coupled. Yet, we could also speculate that they may represent regulative couplings, i.e. that all these structures might have been affected by a single regulatory genetic system.

It is interesting to note that fossil larval representatives of Berothidae possess a higher number of palp elements than any known larva of a modern representative of Berothidae [53]. The new larva could therefore not necessarily exhibit a specialised condition concerning the subdivision of the labial palp, but reflect a more ancestral state with an originally higher number of elements, reduced in various extant lineages. Yet, also this notion remains speculative and depends on more findings of further fossil larval forms of various neuropteran lineages.

### Diversity during the Cretaceous

Many major lineages of Neuroptera appear to have been present in the Cretaceous [64, 66–73]. Many of the specialised larval forms also seem to have been present [16, 52]. Even such highly specialised life styles as the spider-associated, partly parasitic behaviour of larvae of many modern mantis lacewings had most likely already evolved at that time [21].

Yet, the Cretaceous has already provided us with very unusual appearing neuropteran larvae that have no direct counterpart in the modern fauna, but either represent peculiarities or show very unusual combinations of characters [16–18, 52, 74]. The new larva also represents such a case. This observation supports a pattern recognised by Haug et al. [75], who reported that the Mesozoic has seen larval forms of achelatan crustaceans that disappear afterwards, without a necessary loss of diversity in the taxonomic sense. The absence of these specific larvae in later faunas indeed represents a loss, in the sense that the ecological role of these larvae is no longer present. Similarly, the new larva with its distinct morphology might have fulfilled an ecological role absent in later faunas. At least, we know that we have lost this peculiar morphology.

## Conclusions

Despite the fact that a phylogenetic interpretation of the new larval specimen is difficult, it represents a so far unknown morphology that was present in the Cretaceous fauna but is now extinct. Our approach to use body measurements for characterising such taxonomically complicated fossils provided a helpful guideline in this case. Especially for fossils of immature individuals, which can often not be determined to species level (or even to larger groups), this type of approach will allow to integrate these into studies on biodiversity of past ecosystems.

## Data Availability

Data sharing is not applicable to this article as no additional datasets were generated or analysed during the current study.
